# Creatine transporter (SLC6A8) knockout mice exhibit reduced muscle performance, disrupted mitochondrial Ca^2+^ homeostasis, and severe muscle atrophy

**DOI:** 10.1038/s41419-025-07381-x

**Published:** 2025-02-14

**Authors:** Irene Pertici, Donato D’Angelo, Denis Vecellio Reane, Massimo Reconditi, Ilaria Morotti, Elena Putignano, Debora Napoli, Giorgia Rastelli, Gaia Gherardi, Agnese De Mario, Rosario Rizzuto, Simona Boncompagni, Laura Baroncelli, Marco Linari, Marco Caremani, Anna Raffaello

**Affiliations:** 1 PhysioLab (Department of Biology and Department of Experimental and Clinical Medicine), University of Florence, Florence, Italy; 2 Department of Biomedical Sciences, University of Padova, Padova, Italy; 3 Institute for Diabetes and Obesity, Helmholtz Diabetes Center, Helmholtz Zentrum Munich, Munich, Germany; 4 Institute of Neuroscience, National Research Council (CNR), Pisa, Italy; 5 Center for Advanced Studies and Technology, Department of Neuroscience, Imaging, and Clinical Sciences, University G. D’Annunzio of Chieti-Pescara, Chieti, Italy; 6 National Center of Gene Therapy and RNA-based Drugs, Padova, Italy; 7 Department of Developmental Neuroscience, IRCCS Stella Maris Foundation, Pisa, Italy; 8 Myology Center (CIR-Myo), University of Padua, Padua, Italy

**Keywords:** Physiology, Pathogenesis, Mitochondria

## Abstract

Creatine (Cr) is essential for cellular energy homeostasis, particularly in muscle and brain tissues. Creatine Transporter Deficiency (CTD), an X-linked disorder caused by mutations in the SLC6A8 gene, disrupts Cr transport, leading to intellectual disability, speech delay, autism, epilepsy, and various non-neurological symptoms. In addition to neurological alterations, Creatine Transporter knockout (CrT^−/y^) mice exhibit severe muscle atrophy and functional impairments. This study provides the first characterization of the skeletal muscle phenotype in CrT^−/y^ mice, revealing profound ultrastructural abnormalities accompanied by reduced fiber cross-sectional area and muscle performance. Notably, mitochondria are involved, as evidenced by disrupted cristae, increased mitochondrial size, impaired Ca^2+^ uptake, reduced membrane potential and ATP production. Mechanistically, the expression of atrophy-specific E3 ubiquitin ligases and suppression of the IGF1-Akt/PKB pathway, regulated by mitochondrial Ca^2+^ levels, further support the atrophic phenotype. These findings highlight the profound impact of Cr deficiency on skeletal muscle, emphasizing the need for targeted therapeutic strategies to address both the neurological and peripheral manifestations of CTD. Understanding the underlying mechanisms, particularly mitochondrial dysfunction, could lead to novel interventions for this disorder.

## Introduction

Creatine (Cr) plays a crucial role in regulating cellular energy homeostasis, particularly in high-demanding tissues such as the muscle and the brain [1]. Creatine Kinase (CK) converts Cr into phospho-creatine (PCr), a metabolically inert phosphagen used for temporal and spatial buffering of ATP levels. The PCr/CK system is vital for high-intensity physical exercise in striated muscle and heart contraction when ATP hydrolysis exceeds production by other metabolic pathways [2, 3].

Approximately one-half of the daily Cr needs in humans comes from the diet, with the remaining one-half synthesized endogenously through a two-step enzymatic pathway involving L-arginine:glycine amidinotransferase (AGAT) and S-adenosyl-L-methionine:N-guanidinoacetate methyltransferase (GAMT) mostly in the liver, pancreas, and kidneys [3, 4]. After synthesis or nutritional supply, Cr is released into the bloodstream to nourish all body cells. However, Cr cannot cross the lipid membrane and requires a specific Na^+^/Cl^−^-dependent transporter (Cr transporter, CrT). In humans, CrT is predominantly expressed in the muscle, kidney, heart, and other tissues, including the brain [5–7].

Creatine Transporter Deficiency (CTD, OMIM #300352) is a monogenic X-linked disorder [8], associated with multiple mutations in the solute carrier family 6-member 8 (Slc6a8) gene that encodes the protein responsible for cellular Cr transport [8, 9]. Recent studies suggest that the prevalence of this disorder might range between 1% and 3% in males with intellectual disability [8, 10]. However, the lack of prenatal and perinatal screening makes CTD a still underdiagnosed disorder. Clinical hallmarks include intellectual disability, severe speech delay, autistic features, epilepsy, and movement disorders [9]. Patients also experience non-neurological symptoms, including gastrointestinal dysfunction, bladder dysfunction, cardiomyopathy, and ophthalmologic abnormalities [11, 12]. Skeletal muscle mass reduction and mild muscle weakness have been described [12, 13], primarily as late onset deficits [14–16]. While Cr concentration in skeletal muscle appears to be within the normal range, this has only been analyzed for a few specific mutations [17, 18].

The absence of a functional CrT renders Cr supplementation ineffective for CTD [8]. Consequently, effective treatments for CTD remain elusive, posing ongoing challenges for patients and their families.

To understand the causative mechanisms and the main molecular pathways altered in CTD, animal models lacking the Slc6a8 gene or engineered to express an allele with the point mutation found in patients have been generated [19–22]. These rodent lines have significantly contributed to dissecting the pathological determinants of this disorder [8]. They revealed that the loss-of-function of Slc6a8 does not result in overt alterations in brain structure and neuronal density but rather in a subtle reorganization of cerebral circuits and cellular metabolic processes [19, 22–24]. However, the cellular and molecular players involved in the development and progression of the CTD phenotype, not only in the brain but also in skeletal muscle, remain largely unexplored [8].

Interestingly, unlike CTD patients, skeletal muscles of CrT knockout (CrT^−/y^) animals exhibit low Cr levels and a severely compromised phenotype, characterized by impaired motor function, muscle atrophy, increased glucose metabolism due to AMPK activation [21, 25], and susceptibility to fatigue [26]. Motor alterations are absent in mice harboring a brain-specific Slc6a8 deletion, suggesting a peripheral contribution to the onset of CTD pathology [27, 28]. Nevertheless, a complete description of the skeletal muscle phenotype and the underlying mechanisms remain elusive. The study of this aspect is particularly important given that the PCr-CK system requires high levels of total Cr (20-40 mM) in skeletal muscle [29] that can be achieved only with functional cellular Cr uptake [29].

Interestingly, CTD clinical phenotype closely resembles that observed in a wide range of mitochondrial disorders (e.g., developmental, or cognitive disabilities, seizures, fatigue and cardiomyopathy). CTD patients initially suspected of having a mitochondrial disorder were later found to have a creatine transporter defect. Notably, there is a strong link between Cr and mitochondrial function, and changes in PCr/Cr ratio finely regulate mitochondrial respiration [1, 30–32]. It is thus not surprising that the metabolic phenotype of Cr deficiency results in disturbed metabolic activity, as well as structural abnormalities of mitochondrial organelles [24, 28, 33, 34]. Nevertheless, mitochondrial abnormalities and their causative role in the pathogenesis of this disease have scarcely been assessed at functional and molecular levels. This is a crucial aspect, as preclinical evidence suggests that mitochondria might be promising targets for therapeutic intervention in various brain and motor dysfunctions.

In this study, we describe the profound impact of Cr deficiency on the skeletal muscle of CrT^−/y^ animals. Specifically, we found that muscles exhibit an atrophic phenotype accompanied by fibers ultrastructural alterations, diminished performance, and increased expression of key E3 ubiquitin ligases associated with the progression of atrophy. Additionally, mitochondria display significant morphological abnormalities, reduced membrane potential, and impaired mitochondrial Ca^2+^ uptake, accompanied by changes in the expression of proteins involved in mitochondrial Ca^2+^ homeostasis.

## Results

### CrT^−/y^ muscles exhibit pronounced fiber atrophy and ultrastructural alterations

**Fig. 1:**
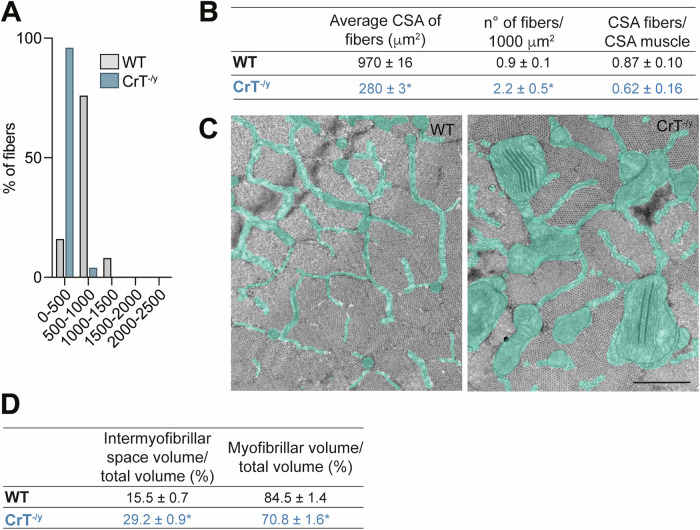
Quantification of the amount of contractile material in EDL muscle from WT and CrT^−/y^ mice. **A** Distribution of the cross-sectional area of the fibers in the EDL muscle of WT and CrT^−/y^ mice. Samples size: 3 EDLs for each group. **B** Cross-sectional area occupied by the fibers within the muscle in WT and CrT^−/y^ mice. **C** Representative electron micrographs of cross sections of EDL fibers from WT (left panel) and CrT^−/y^ (right panel) muscles. The intermyofibrillar space and the myofibrillar volume over the fiber volume are reported (**D**). Scale bar in (**C**): 1 μm. In (**B**) and (**D**), data are presented as mean ± SEM. n = 15. For data analysis, parametric Student t-test (two tailed, unpaired) was used. *p < 0.05.

The mouse model carrying the ubiquitous deletion of 5–7 exons in the Slc6a8 gene (CrT knockout, CrT^−/y^) [20] exhibits low Cr levels in the brain, skeletal and cardiac muscles, and kidneys [20, 23]. Given that no comprehensive characterization of skeletal muscle structure has been performed in this model thus far and considering the crucial role of Cr in muscle metabolism, we began by assessing the presence of qualitative and quantitative ultrastructure alterations. Using Transmission Electron Microscopy (TEM), we analyzed the Extensor Digitorum Longus (EDL) muscle in WT and CrT^−/y^ mice at Post Natal Day (PND) 40. In WT mice, the average cross-sectional area (CSA) of the fibers is 970 ± 16 μm^2^ (mean ± SEM) (Fig. 1A, *gray columns* and Fig. 1B), while in CrT^−/y^ mice the CSA of the fibers is significantly reduced to 280 ± 3 μm^2^ (Fig. 1A*blue columns and* Fig. 1B). The number of fibers per 1000 μm^2^ is 0.9 ± 0.1 in WT mice, resulting in a fractional area of the muscle occupied by the fibers of [(970 μm^2^/fiber)/(1000 μm^2^/0.9 fibers)]= 0.87 ± 0.10 (Fig. 1B). In CrT^−/y^ mice, with 2.2 ± 0.5 fibers per 1000 μm^2^ (Fig. 1B), the fractional area is the muscle occupied by the fibers is 0.62 ± 0.16 (Fig. 1B), approximately 30% lower than in WT mice. CrT^−/y^ muscles exhibit an increased fraction of intermyofibrillar space relative to fiber volume (0.292 ± 0.009) compared to WT muscles (0.155 ± 0.007), resulting in a decreased fractional fiber volume (0.708 ± 0.016 in CrT^−/y^ mice and 0.845 ± 0.014 in WT mice, Fig. 1C, D). The CSA occupied by the myofibrils within the muscle can be determined multiplying the CSA of the EDL muscle, estimated from the wet weight of the muscle (see Methods), by a factor *α* = 0.87 × 0.845 = 0.74 ± 0.08 (or 74%) for the WT mice and 0.62 × 0.708 = 0.44 ± 0.10 (or 44%) for the CrT^−/y^ mice.

### Muscle performance impairments and myosin heavy chain isoform shifts in CrT-/y mice

**Fig. 2:**
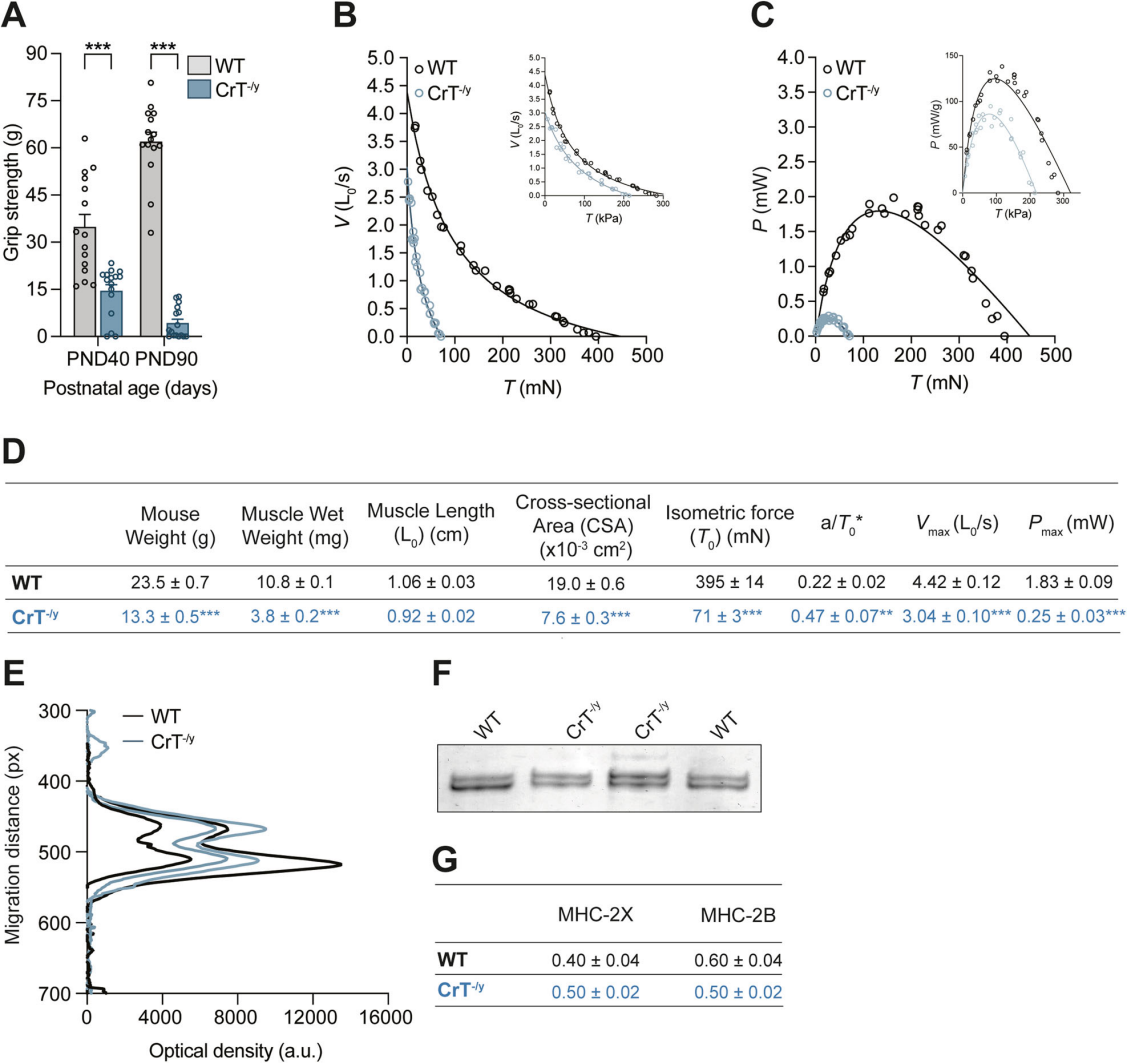
Grip strength test, force-velocity relation, power output, and myosin heavy chain expression in EDL muscles of WT and CrT^−/y^ mice. **A** Grip strength test was performed on postnatal day (PND) 40 and PND90. For data analysis, two-way ANOVA, post hoc Holm-Sidak. Data from n = 12. **B** Relation between force (*T*, mN) and shortening velocity (*V*, *L*_0_/s) in WT (black symbol) and CrT^−/y^ (blue symbol) mice. Lines are Hill’s hyperbolic equations fit to the data (same color codes for data). Inset, same relations with force expressed in kPa after correction of the muscle CSA for the myofibrillar density in the muscle (factor α, see Text). **C** Power-force relations calculated from data (symbols) and their fits (lines) in B. Inset, same relations with force expressed in kPa after correction of CSA for the factor α. **D** Mechanical parameters during isometric contraction and during isotonic shortening of muscles from WT and CrT^−/y^ mice. *T*_0_, maximal isometric force; a/*T*_0_^*^, is a measure of the curvature of the force–velocity relation; *V*_max_, unloaded shortening velocity; *P*_max_, maximum power output. *T*_0_^*^ is the intercept of the Hill equation on the force-axis. In panel D, data are presented as mean ± SEM. n = 4. **E** and **F** MHC isoform identification of the EDL muscle fibers by SDS-PAGE in the area of migration of the myosin heavy chains (~220 kDa). In (E) are shown the projections of the mass density along the vertical axis of the bands in (F) after horizontal integration. **G** Fractional expression of the MHC 2X and 2B isoforms for the WT and CrT^−/y^ muscles. For data analysis, parametric Student *t*-test (two-tailed, unpaired) was used. *p < 0.05; **p < 0.01; ***p < 0.001. Temperature 23 °C.

We then investigated whether the observed morphological alterations affected muscle performance. Consistent with the structural changes, grip strength in CrT^−/y^ mice showed a significant reduction, with strength diminished to 45% and 10% of that in WT mice at PND 40 and 90, respectively (Fig. 2A). Additionally, the isometric force (*T*_0_) of EDL muscles from PND40 CrT^−/y^ mice was substantially lower, measuring 71 ± 3 mN, which is just 18 ± 1% of the WT mice’s force (395 ± 14 mN, Fig. 2D).

To further assess muscle performance, we analyzed the force-velocity (*T*-*V*) and the power-force (*P*-*T*) relations. The shortening velocity (*V*), measured following a drop in force from *T*_0_ to a pre-set value *T* lower than *T*_0_ (Supplementary Fig. 1A), was significantly reduced for *T* < 0.4*T*_0_ in CrT^−/y^ mice compared with WT mice (Supplementary Fig. 1B). This reduction was accompanied by a decrease in the curvature of the *T*-*V* relation (increase in *a*/*T*_0_^*^, see Methods and Fig. 2), approximately two-fold, and a reduction in the unloaded shortening velocity (*V*_max_) from 4.4 ± 0.1 L_0_/s in WT mice to 3.0 ± 0.1 L_0_/s in CrT^−/y^ mice, with L_0_ representing the initial muscle length (Fig. 2B, D, and Supplementary Fig. 1B). Power output (*P* = *T* × *V*) at any *T* was lower in CrT^−/y^ mice compared to WT mice (Fig. 2C). The maximum power output (*P*_max_), achieved at about one-third of the maximum force, was reduced to approximately 14% of WT levels (from 1.83 ± 0.09 mW to 0.25 ± 0.03 mW, Fig. 2C, D).

The reduced muscle performance could be linked to a shift of the myosin heavy chain (MHC) isoforms expressed in the EDL muscle. The MHC-2X:MHC-2B ratio is 2:3 in the WT mice and 1:1 in the CrT^−/y^ mice with a shift in the expression of the faster MHC-2B isoform to the slower MHC-2X isoform (Fig. 2E–G). Considering that fibers expressing pure MHC-2B and MHC-2X isoforms have similar isometric force while the unloaded shortening velocity of the fibers expressing the MHC-2X isoform is about ½ of those expressing the MHC-2B isoform [35], the lower *V*_max_ found in the EDL muscle of mutated mice could be explained by the increased expression of the slower MHC-2X isoform with respect to the faster MHC-2B isoform. However, the shift in the MHC isoforms alone would not fully account for the reduction in *P*_max_ (Supplementary Fig. 1C). Therefore, other factors, such as fiber atrophy, structural alterations of sarcomeres, or altered Ca^2+^ handling, must contribute to the reduced muscle performance.

**Fig. 3:**
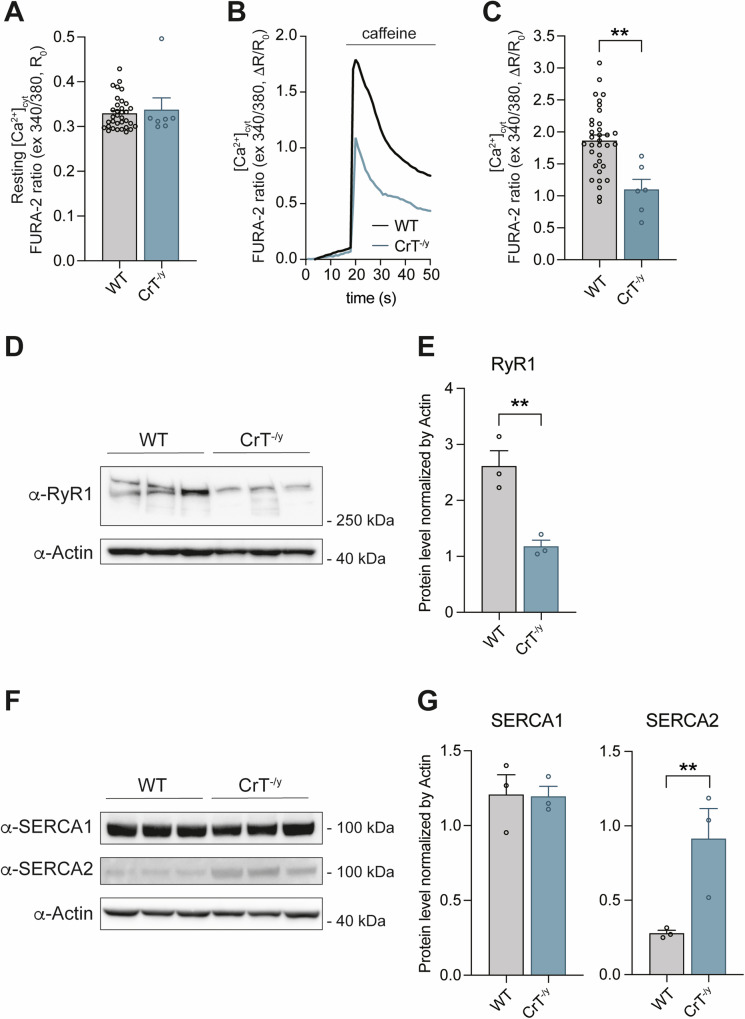
Characterization of cytosolic Ca^2+^ homeostasis in FDB fibers of WT and CrT^−/y^ mice. WT and CrT^−/y^ myofibers were loaded with cytosolic Fura-2/AM. **A** Resting cytosolic Ca^2+^ concentrations; **B** representative traces of cytosolic Ca^2+^ concentrations upon caffeine stimulation; **C** cytosolic Ca^2+^ concentrations upon caffeine stimulation. Data are presented as mean ± SEM. n > 20. For data analysis, parametric Student t-test (two-tailed, unpaired) was used. ** p < 0.01. **D** Representative Western blot of WT and CrT^−/y^ TA muscles stained with α-RyR1 antibody. α-Actin was used as loading controls. n = 3. **E** Quantification of the immunoblots showed in D. The levels of the protein were normalized by actin levels. Data are presented as mean ± SEM. n = 3. For data analysis, parametric Student *t*-test (two-tailed, unpaired) was used. ** p < 0.01. **F** Representative Western blot of WT and CrT^−/y^ TA muscles stained with α-SERCA1 and α-SERCA2 antibodies. α-Actin was used as loading controls. n = 3. **G** Quantification of the immunoblots showed in D. The levels of the protein were normalized by actin levels. Data are presented as mean ± SEM. n = 3. For data analysis, parametric Student *t*-test (two-tailed, unpaired) was used. **p < 0.01.

Focusing on Ca^2+^ dynamics, we measured cytosolic Ca^2+^ concentration [Ca^2+^]_cyt_ on isolated Flexor Digitorum Brevis (FDB) fibers upon caffeine stimulation using Fura-2/AM. While resting [Ca^2+^]_cyt_ was unaffected (Fig. 3A), caffeine-induced Ca^2+^ transients were significantly reduced in CrT^−/y^ mice compared to WT (Fig. 3B, C), suggesting either a lower amount of Ca^2+^ released from the sarcoplasmic reticulum (SR) or a reduced capacity of the mitochondrial uptake machinery. To analyze changes observed in cytosolic Ca^2+^ homeostasis, we examined whether the expression of the SR release channel RyR1 and the uptake pumps SERCA1 and SERCA2 were affected by the KO of CrT. Consistent with the reduction in cytosolic Ca^2+^ levels upon caffeine stimulation, protein levels of RyR1 are significantly reduced (Fig. 3D, E). Concurrently, SERCA2 levels, but not SERCA1 levels, are increased (Fig. 3F, G).

### Mitochondria of CrT^−/y^ fibers are structurally altered, with reduced membrane potential and Ca^2+^ uptake capability

**Fig. 4:**
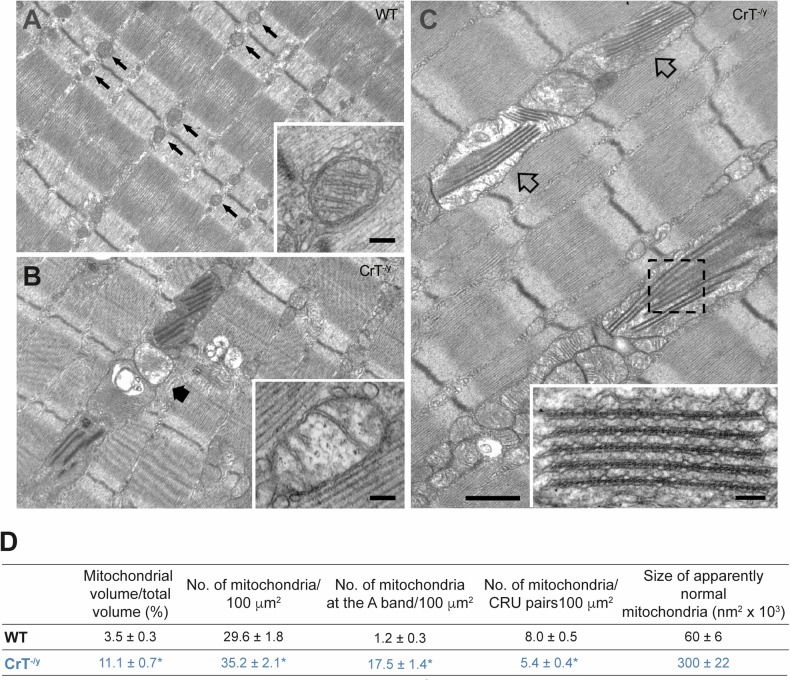
CrT^−/y^ mitochondria present morphological abnormality. Representative electron microscopy pictures of EDL muscle fibers in control (**A**) and CrT^−/y^ mice (**B**) and (**C**). Scale bars: 1 μm (insets: 0.1 μm). **D** Quantitative EM analysis. Data are presented as mean ± SEM. Sample size: 15 fibers from 1 WT mice, 30 fibers from 2 CK-KO mice; 5 micrographs/fiber. For data analysis, parametric Student *t*-test (two-tailed, unpaired) was used. *p < 0.05.

Given the well-established link between Cr and mitochondrial function [24, 28, 36], we investigated mitochondrial organization and morphology in EDL fibers of CrT^−/y^ mice at PND40. In adult EDL fibers of WT mice, mitochondria are predominantly located at the I band on both sides of the Z-line (Fig. 4A, *small arrows*) [37] are rarely found at the A-band of the sarcomere (Fig. 4A, D). These mitochondria exhibit a round or oval shape, with an average area of 60 ± 6 ×10^3^ nm^2^ (Fig. 4D) and display parallel cristae within a dense matrix (Fig. 4A, *inset*). They occupy about 3.5% of the total cell volume, with 8.0 ± 0.5 per 100 μm^2^ of triads pairs (i.e. the Ca^2+^ release units, CRUs) (Fig. 4D). In contrast, CrT^−/y^ EDL fibers show a pronounced alteration in mitochondrial distribution. A large number of mitochondria are aligned along the A-band, forming longitudinal columns between myofibrils (Fig. 4B, *large arrow*), with 17.5 ± 1.4 mitochondria per unit area at the A band (Fig. 4D). These mitochondria are five-time larger (300 ± 22 × 10^3^ nm^2^), occupy a significantly larger fiber volume (11.1 ± 0.7%), and are less frequently associated with CRUs (5.4 ± 0.4 mitochondria per CRU pairs per unit area) (Fig. 4D). Additionally, several mitochondria display structural abnormalities, including a clear matrix and damaged internal cristae (indicated by a large black arrow in Fig. 4B, *and inset*). Moreover, damaged mitochondria frequently contain multi-layered structures (Fig. 4C, *empty arrows*), resembling lamellae arranged in a regular fashion, which may significantly alter the internal cristae organization (Fig. 4C, *dashed box and inset*). Other notable structural alterations include: i) a high incidence of fibers containing lipid droplets (Supplementary Fig. 2), which are typically absent in WT EDL fibers, and ii) about 35% of fibers showing severe damage, characterized by a total loss of cross-striation and complete disruption of the sarcomeric structure.

**Fig. 5:**
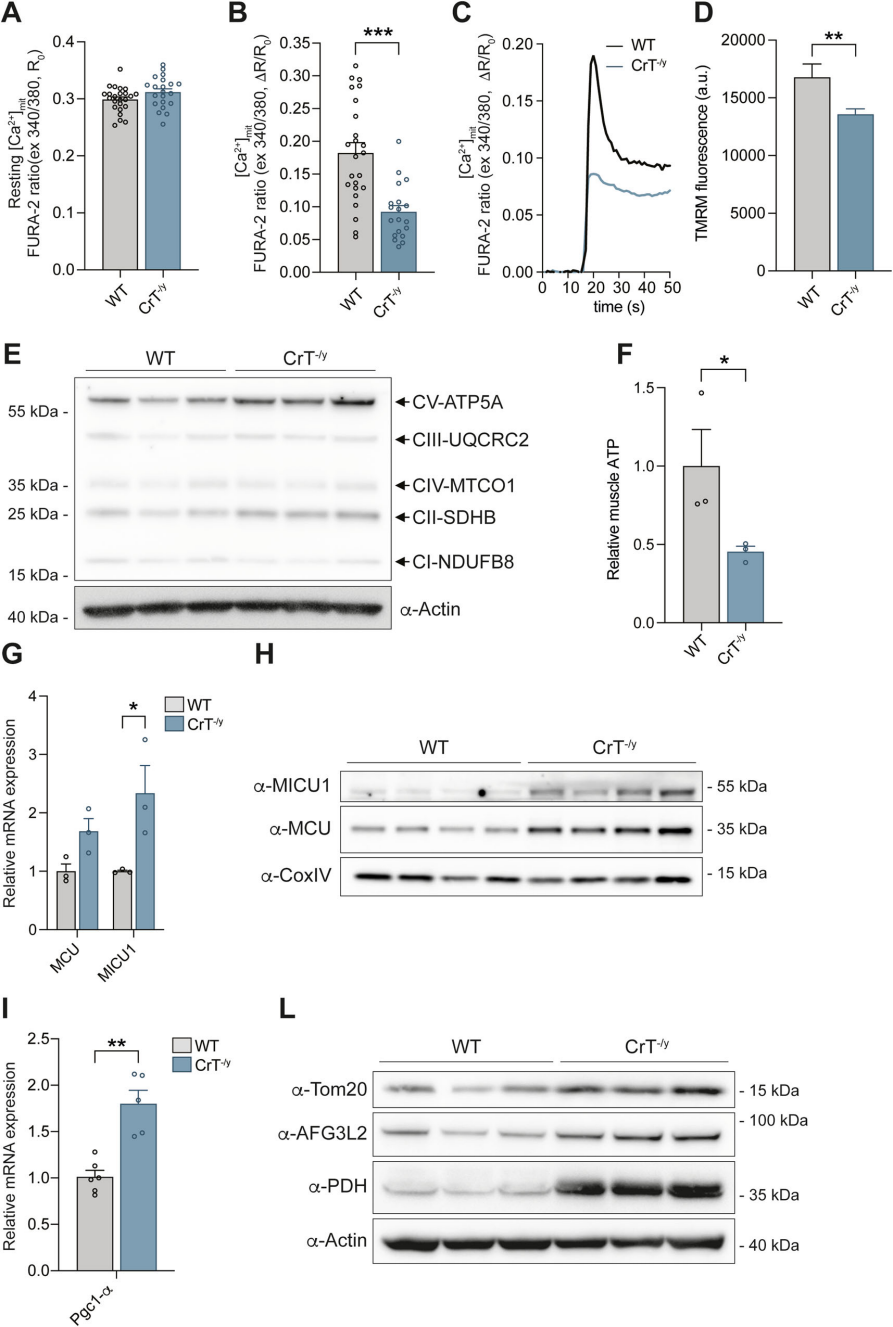
Mitochondrial Ca^2+^ uptake and membrane potential are decreased in CrT^−/y^ muscles compared to WT and the mRNA and protein expression of crucial components of the MCU complex is elevated. WT and CrT^−/y^ myofibers were loaded with mitochondrially targeted Fura-2/AM (mitoFura-2/AM). **A** Resting mitochondrial Ca^2+^ concentrations are unaltered in CrT^−/y^ myofibers compared to WT. **B** Ratiometric measurements of mitochondrial Ca^2+^ uptake upon caffeine treatment in CrT^−/y^ FDB myofibers compared to WT fibers. **C** Representative traces of the experiment. Data are presented as means ± SEM. n > 20. For data analysis, parametric Student *t*-test (two-tailed, unpaired) was used. ***p < 0.001. **D** TMRM fluorescence in FDB fibers of WT and CrT^−/y^ mice. Data are presented as mean ± SEM. n = 20. For data analysis, parametric Student *t*-test (two-tailed, unpaired) was used. **p < 0.01. **E** Representative Western blot of the OXPHOS complexes in WT and CrT^−/y^ muscles. n = 3. **F** Relative skeletal muscle ATP levels in EDL muscles of WT and CrT^-/y^ muscles. n = 3. For data analysis, parametric Student *t*-test (two-tailed, unpaired) was used. *p < 0.05. **G** Real-time RT-PCR analyses of WT and CrT^−/y^ muscles with the indicated oligonucleotide primers. Data are presented as mean ± SEM. n = 3. For data analysis, parametric Student *t*-test (two-tailed, unpaired) was used. *p < 0.05. **H** Representative Western blot of WT and CrT^−/y^ EDL muscles. α-CoxIV was used as loading control. n = 4. **I** Real-time RT-PCR analyses of WT and CrT^−/y^ muscles with the indicated oligonucleotide primers. Data are presented as mean ± SEM. n = 5. For data analysis, parametric Student *t*-test (two-tailed, unpaired) was used. **p < 0.01. **L** Representative Western blot of WT and CrT^−/y^ TA muscles stained with α-Tom20, α-AFG3L2 and α-PHD antibodies. α-Actin was used as loading controls. n = 3.

Mitochondrial impairment extends beyond the structural level. Specifically, by using mitochondrially targeted Fura-2/AM (mitoFura-2/AM) we found that while basal mitochondrial Ca^2+^ concentrations ([Ca^2+^]_mt_) were comparable between WT and CrT^−/y^ FDB muscle fibers (Fig. 5A), CrT^−/y^ fibers exhibited a significant reduction in mitochondrial Ca^2+^ uptake following caffeine-induced Ca^2+^ release from the SR (Fig. 5B, C). We then investigated whether the observed Ca^2+^ phenotypes might reflect changes in the driving force of mitochondrial Ca^2+^ accumulation. To assess this, we measured mitochondrial membrane potential (ΔΨ_m_) using tetramethyl rhodamine methyl ester dye (TMRM) in non-quenching mode, wherein the dye is applied at low concentrations to avoid aggregation and quenching within mitochondria. As a result, depolarized mitochondria accumulate less of the cationic dye, resulting in lower fluorescence, while hyperpolarized mitochondria show increased fluorescence due to higher dye accumulation [38]. CrT^−/y^ mice showed a significant decrease in ΔΨ_m_, indicating potential impairment in ATP production (Fig. 5D). As mitochondrial Ca^2+^ plays a well-established role in regulating oxidative metabolism and the ΔΨ_m_ is the driving force for ATP production [39], we analyzed whether CrT^−/y^ is characterized by alteration of electron transport chain. Using Western blot analysis with a Total OXPHOS Antibody Cocktail kit, targeting specific subunits of the five OXPHOS complexes (NDUFB8, SDHB, UQCRC2, MTCO1, and ATP5A), we found that CrT deletion does not reduce the protein levels of these OXPHOS subunits. Instead, a slight increase was observed in complexes V and II, indicating the possible activation of potential compensatory mechanisms (Fig. 5E). Further analysis of ATP production in muscle tissue, indeed, showed that CrT deletion leads to a significant reduction in ATP levels (Fig. 5F), supporting an overall impairment in mitochondrial function in CrT^−/y^ muscles. Given the reduced mitochondrial Ca^2+^ entry, we investigated the expression of the different components of the Mitochondrial Calcium Uniporter (MCU) complex, the channel responsible for Ca^2+^ entry into mitochondria. Our results revealed a significant increase in the mRNA expression of the crucial regulator of the channel MICU1 (Fig. 5G), as well as elevated protein levels of both the pore-forming subunit MCU and MICU1 in CrT^−/y^ mice compared to WT (Fig. 5H). To explore the discrepancy between reduced mitochondrial Ca^2+^ uptake and increased expression of MCU complex components, we measured Ca^2+^ extrusion rates in WT and CrT^−/y^ muscles. We found no significant differences between the two groups (Supplementary Fig. 3). Furthermore, analysis of the mRNA expression of the Pgc1-α gene showed that the lack of CrT triggers the induction of Pgc1-α (Fig. 5I), indicating enhanced mitochondriogenesis, which aligns with our ultrastructural findings (Fig. 4) and the increased expression of MCU complex subunits. We also analyzed the protein expression of key mitochondrial markers from distinct compartments: Tom20 (outer membrane), AFG3L2 (inner membrane), and PDH (matrix). The expression levels of all these proteins were elevated in CrT^−/y^ muscles compared to WT (Fig. 5L and Supplementary Fig. 4), further corroborating our previous findings of mitochondrial alterations. In summary, these findings suggest that CrT deficiency in skeletal muscle leads to compromised mitochondrial Ca^2+^ homeostasis, which may contribute to the observed pathological phenotype.

### The IGF-1/Akt/PKB pathway is suppressed and the expression of atrophy-specific E3 ubiquitin ligases is induced in CrT^−/y^ mice

**Fig. 6:**
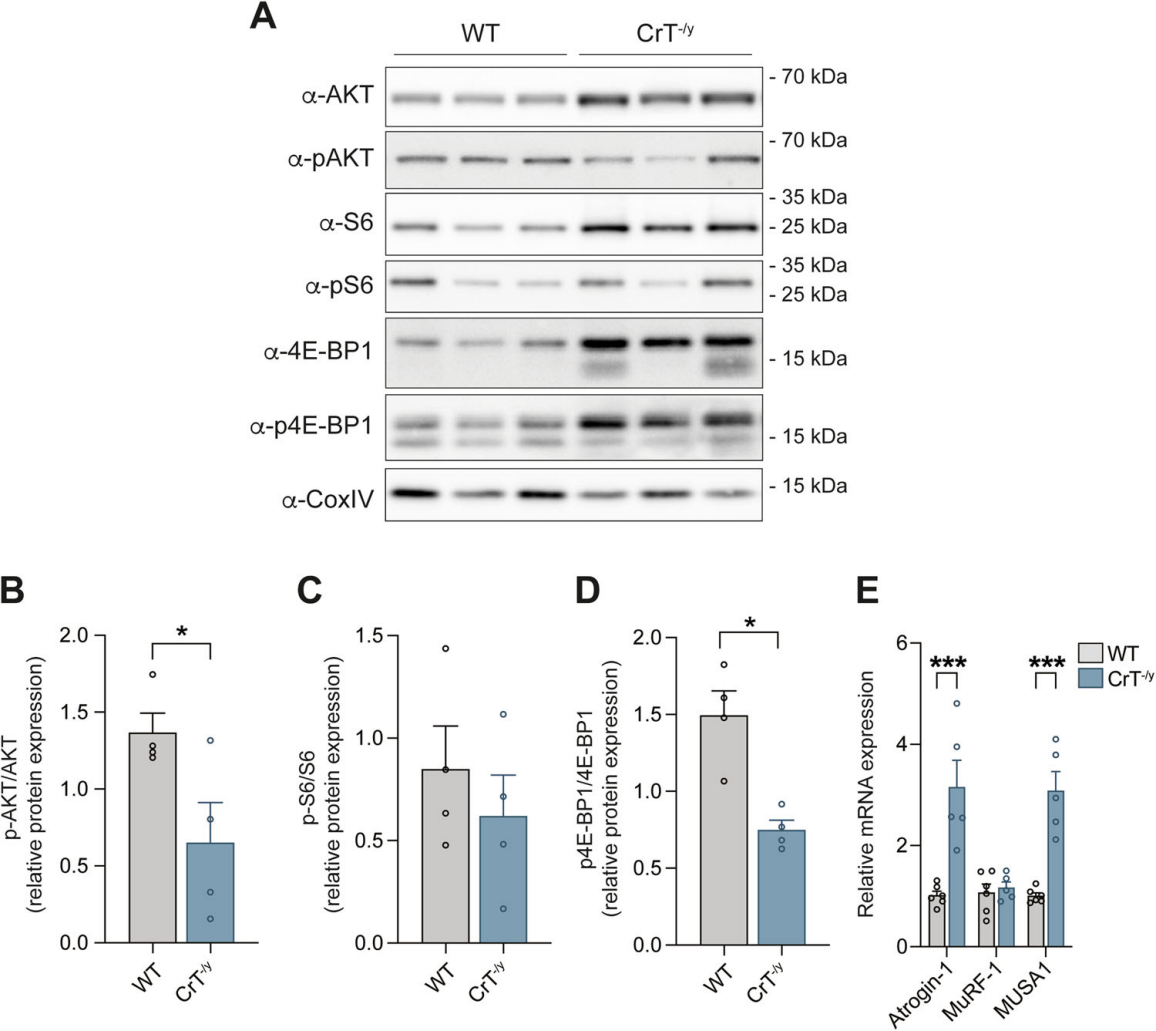
The lack of CrT induces mitochondriogenesis and specific atrophy programs. **A** Representative Western blot of WT and CrT^−/y^ EDL muscles stained with the indicated antibodies. α-CoxIV was used as loading control. n = 3. **B**–**D** Quantification of the experiment performed as in (A). Data are presented as mean ± SEM. n = 4. For data analysis, parametric Student t-test (two-tailed, unpaired) was used. *p < 0.05. **E** Real-time RT-PCR analyses of WT and CrT^−/y^ muscles with the indicated oligonucleotide primers. Data are presented as mean ± SEM. n = 5. For data analysis, parametric Student t-test (two-tailed, unpaired) was used. ***p < 0.001.

To elucidate the mechanism underlying the atrophic phenotype in CrT^−/y^ animals, we directed our focus toward the established hypertrophy pathways in skeletal muscle, specifically the IGF1-Akt/PKB signaling axis [40]. This pathway is crucial for promoting muscle hypertrophy, whereas its suppression is known to lead to muscle atrophy [40]. In CrT^−/y^ animals, we observed a marked suppression of Akt signaling, as evidenced by reduced phosphorylation of Akt, S6, and 4E-BP1 (Fig. 6A–D). These findings suggest that the atrophic phenotype in CrT^−/y^ mice is likely due to impaired IGF1/Akt/PKB signaling. Additionally, we noted a significant upregulation of Atrogin-1 and MUSA1, two E3 ubiquitin ligases known to be involved in protein degradation during muscle atrophy [40] (Fig. 6E). This increased expression further supports the association between CrT deficiency and enhanced muscle atrophy through disrupted signaling pathways and elevated protein degradation.

## Discussion

Creatine (Cr) is essential for supplying cells with high-energy phosphates, facilitating the rapid regeneration of hydrolyzed ATP. The creatine transporter (CrT/SLC6A8) is crucial for transporting Cr across tissue barriers and into specific cells, such as neurons and myocytes [41]. Mutations in the CrT gene lead to Creatine Transporter Deficiency (CTD), associated with severe intellectual disability, epilepsy, autism, developmental delay, and motor dysfunction [8, 41]. While these manifestations primarily impact brain function, non-neurological symptoms, including gastrointestinal dysfunction, bladder dysfunction, cardiomyopathy, and ophthalmologic abnormalities, are also observed [11, 12]. Skeletal muscle hypotonia and nonspecific myopathic symptoms are common, though the literature on muscle Cr levels in CTD patients is poor [13, 17, 18]. Despite the limited understanding of peripheral contributions to CTD pathology, evidence suggests that muscle impairment could affect neurological symptoms, as brain-specific CrT^−/y^ mice do not fully reproduce the early pathological features or autistic traits of CTD patients [42].

To address the gap in understanding muscle phenotype, we characterized the skeletal muscle of the CrT^−/y^ mouse model, which exhibits reduced body weight and cognitive impairments similar to those observed in CTD patients [23]. Creatine levels in the skeletal muscle of these mice were dramatically reduced (~65% at PND30 and ~ 95% in adults, [23]), with insufficient compensation from increased AGAT expression [21, 43]. Our data clearly show that CrT^−/y^ muscles are dramatically atrophic (Fig. 1A, B) in accordance with previous results [21, 25]. Additionally, in EDL muscles the proportion of contractile elements in terms of fiber volume is significantly diminished in CrT^−/y^ muscles compared to WT, leading to a consequent increase of the intermyofibrillar space (Fig. 1C, D). This observation supports the atrophic phenotype of this murine model. Fiber atrophy in CrT^−/y^ muscles is associated to a functional decline, characterized by reduced grip strength, both at PND 40 and 90, with a marked reduction in isometric force and power at PND 40 (Fig. 2A–C).

In WT mouse, with a CSA of the EDL muscle of 19 × 10^−3 ^cm^2^ (Fig. 2D), the specific isometric force per unit area is (395 mN / 19 × 10^−3 ^cm^2^ =) 208 ± 10 kPa. As the muscle fibers represent 87% of the cross-sectional area and the area of the fiber occupied by the myofibrils is 84.5% (Fig. 1B), the CSA occupied by the myofibrils is α = 74% of the muscle CSA, and *T*_0_ is (395 mN/(19 × 10^−3 ^cm^2^ × α) = ) 281 ± 10 kPa. In CrT^−/y^ mouse, with a muscle CSA = 7.6 × 10^−3 ^cm^2^ (Fig. 2D) and α = 44% (muscle fibers occupy 62% of the muscle CSA; myofibrils occupy 71% of the fiber CSA, Fig. 1B), the same calculation gives an isometric force of 212 ± 17 kPa, about 30% lower than in WT mouse. After correction, the maximum power developed by the myofibrils within the muscle is about 35% lower in CrT^−/y^ mouse (82 ± 2 mW/g) than in control (128 ± 2 mW/g) (*Inset* Fig. 2C). The reduced power in CrT^−/y^ mice cannot be explained by differences in mechanical parameters resulting from the shift in myosin heavy chain isoforms (Supplementary Fig. 1C). Instead, it may be related to the disruption of sarcomere structure observed in some fibers (Supplementary Fig. 2), a decreased cytosolic [Ca^2+^] in response to caffeine (Fig. 3B, C), reduced Ca^2+^ release from the ER, as indicated by the lower protein levels of RyR1 (Fig. 3D, E), and an increased capacity of the SR to reuptake Ca^2+^ upon stimulation, evidenced by elevated SERCA2 protein levels (Fig. 3F, G). Furthermore, in addition to the shift from the faster MHC-2B isoform to the slower MHC-2X isoform, the presence of disrupted sarcomeres behaving as passive elements could also generate an internal load and contribute to the reduction of the shortening velocity more evident at low loads.

Cytoarchitectural abnormalities and a higher proportion of the cell volume occupied by mitochondria, resulting in a decreased concentration of myofibrils, have been observed in other models of creatine deficiency [27, 28, 44] and an increase in [Ca^2+^]_cyt_ occurs only during repeated tetanic stimulation, accompanied by a significant decline in force in CK knockout mouse fibers [26].

The structural analysis of the current study has been focused on EDL muscles. EDLs are mostly composed of type IIb (fast-twitch glycolytic) fibers which rely mainly on glycogen for anaerobic metabolism. Contrary to type I (slow-twitch oxidative) fibers, which have normally a high content of lipid droplets and mitochondria as their energy metabolism is completely aerobic, type IIb fibers have reduced mitochondrial volume and lower contents of lipid droplets [45]. The structural observations and the quantitative analysis revealed that in EDL fibers from CrT^−/y^ mice there is an increase in mitochondrial number/volume and size (Fig. 4D), accompanied by a corresponding increase in the protein expression levels of mitochondrial outer and inner membrane and matrix proteins (Fig. 5L and Supplementary Fig. 4). In addition, in CrT^−/y^ fibers a high percentage of mitochondria is placed at the A-band of the sarcomere, a spatial characteristic disposition of slow-twitch fibers (Fig. 4D). Interestingly, an unusual high number of lipid droplets, which are usually not present in WT EDL fibers, has been also observed (Supplementary Fig. 2). A similar phenomenon has been documented in the AGAT^−/−^ model of creatine deficiency [28]. In this model, the increased activity of citrate synthase and F-type ATPase, along with the presence of intramyocellular lipid droplets, indicates a boost in oxidative ATP production within the mitochondria of resting AGAT^−/−^ muscle [28].

These structural results significantly support the change in the fiber composition and/or metabolism, with a fiber-type shift towards a slower isoform (Fig. 2E–G), induced by the absence of CrT.

In CrT^−/y^ mice, the increased mitochondrial volume is accompanied by elevated expression of the master regulator of mitochondriogenesis, PGC-1α (Fig. 5I), suggesting that creatine deficiency in skeletal muscle induces a precise transcriptional program. This result not only supports the increase in both the quantity and size of mitochondria (Figs. 4, 5L and Supplementary Fig. 4) but also accounts for the slower myosin heavy chain phenotype (Fig. 2F). Indeed, this transcriptional co-activator, working in conjunction with Mef2, activates transcription and acts as a target for calcineurin signaling, a pathway implicated in the expression of genes associated with slow fibers [46]. Overexpression of PGC-1α in skeletal muscle has been shown to induce angiogenesis, improve muscle endurance, motor coordination, and balance in aged animals, and activate mitochondrial oxidative metabolism [46]. Hence, the elevated expression of PGC-1α in CrT^−/y^ may also serve as a compensatory mechanism activated to mitigate atrophy.

Numerous mitochondria in EDL CrT^−/y^ fibers showed a very peculiar structural arrangement of internal cristae resembling multi-layered crystal-like structure rows (Fig. 4C, *empty arrows*). These structures were previously reported in the AGAT^−/−^ model of creatine deficiency [40], but up today either assembling or function is unknown. However, a comprehensive assessment of this issue extends beyond the scope of the current study.

Mitochondrial alterations extend beyond structure to function. Indeed, we observed a significant reduction in ΔΨ_m_ (Fig. 5D) and a marked decrease in Ca^2+^ influx into the mitochondria in isolated fibers of the FDB CrT^−/y^ muscle stimulated with caffeine (Fig. 5B, C). Notably, we did not observe any changes in the quantification of Ca^2+^ under basal conditions (Fig. 5A). Although the protein expression levels of some of the components of the respiratory chain complexes were not reduced but rather induced in CrT^−/y^ muscles compared to WT (Fig. 5E), ATP production is significantly decreased (Fig. 5F). This result aligns with the crucial role of mitochondrial Ca^2+^ in controlling the rate of ATP generation within mitochondria [39].

It is also possible that the observed reduction in mitochondrial Ca^2+^ uptake in isolated fibers of the FDB from CrT^−/y^ mice could at least in part be due to the reduction in the number of CRU-mitochondrial pairs/100 μm^2^ (Fig. 4D) as previously reported in ageing [47] and in SEPN1-related myopathy patients [48, 49].

Specifically, changes in the relationship between mitochondria and CRUs may contribute to impaired delivering of sufficient levels of Ca^2+^ and ATP required for optimal force generation and muscle performance. Data presented here in EDL from CrT^−/y^ mice focus attention on previously unreported alterations not only in mitochondrial morphology but also in their position and orientation with respect to the sarcomeric structure. In EDL fibers from CrT^−/y^ mice, a significantly higher percentage of mitochondria are longitudinally oriented along the A band, rather than occupying their normal I band position (Fig. 4D) and this structural result may likely account for the observed significant reduction (about 35%) in the number of CRU-mitochondria pairs, a factor which could significantly interfere with a proper Ca^2+^ uptake and ATP production.

The decrease in mitochondrial Ca^2+^ is paradoxically accompanied by an elevation in the expression of the pore-forming subunit of the channel responsible for mitochondrial Ca^2+^ entry, MCU, and its positive regulator MICU1 (Fig. 5G, H). This increase, which we have shown to be independent of increased Ca^2+^ extrusion rates from mitochondria (Supplementary Fig. 3), may result from both an expansion of mitochondrial volume and either modifications to other channels (e.g., post-translational modifications) or changes in the expression of other MCU subunits.

We extensively demonstrated that mitochondrial Ca^2+^ entry controls muscle homeostasis [50, 51]. Indeed, silencing MCU triggers muscle atrophy, while its overexpression leads to muscle hypertrophy [51]. In relation to the mechanism, mitochondrial Ca^2+^ uptake regulates skeletal muscle trophism by modulating the IGF-1-AKT/PKB and the PGC-1α4 pathways [51]. Based on these premises, we asked whether signaling pathways controlling muscle homeostasis were altered in CrT^−/y^ animals focusing our attention on the IGF-1/AKT/mTOR pathway, whose activation induces skeletal muscle hypertrophy by stimulating protein translation and concomitantly suppressing protein breakdown by phosphorylating the transcription factor FoxO3 [40]. In line with the marked reduction of the muscle CSA, we found that the phosphorylation levels of AKT and of its downstream target 4E-BP1 are inhibited in EDL muscles of CrT KO mice (Fig. 6A–D). Consistently, the expression of the ubiquitin ligases Atrogin-1 and MUSA1, involved in protein degradation during muscle atrophy [40], are induced (Fig. 6E). The suppression of the IGF-1/Akt/PKB pathway and induction of atrophy-specific E3 ubiquitin ligases, such as Atrogin-1 and MUSA1, further support the link between creatine deficiency and muscle atrophy. While the total knockout model does not differentiate between neuronal and muscular contributions, it is likely that reduced creatine levels in skeletal muscle could impact muscle function, especially under stress conditions like intense exercise or infections.

While employing a total KO model prevents a clear distinction between neuronal and muscular contributions to motor function, and impaired skeletal muscle function may not be a predominant symptom in most CrT-deficient patients, we suggest that diminished creatine levels in skeletal muscle could potentially compromise muscle function, particularly under stress conditions like intense exercise or infections in these patients.

In summary, our findings demonstrate that muscles in CrT^−/y^ mice display significant morphological and functional abnormalities, culminating in a pronounced atrophic phenotype with substantially reduced performance at intermediate-high loads and limited shortening capability at low loads. These findings underscore the role of Cr deficiency in muscle pathology and suggest that muscle impairment may contribute to the overall disease phenotype in CTD. Notably, heterozygous females were not investigated in this study, leaving open questions about whether similar but potentially less severe phenotypes occur in female carriers.

## Materials and methods

### Animals

We employed male mice hemizygous for the deletion of exons 5-7 in the *Slc6a8* gene (KO; CrT^−/y^) and their wild-type (WT; CrT^+/y^) littermates [20]. Animals were raised or acclimatized to the conditions of the animal facility, and housed in a ventilated room under a controlled temperature (21 ± 2 °C), with a 12:12 h light:dark cycle and free access to chow rodent diet and water *ad libitum*.

### Light microscopy

For histological examination by light microscopy (LM) semithin sections (∼700 nm) were cut using a Leica Ultracut R microtome (Leica Microsystem, Vienna, Austria) with a Diatome diamond knife (Diatome, Biel, Switzerland) and were stained in a solution containing 1% toluidine blue O and 1% sodium borate (tetra) in distilled water for 3 min on a hot plate at 55–60 °C. After washing and drying, sections were mounted with DPX media for histology (Sigma–Aldrich, Milan, Italy) and observed with a Leica DMLB light microscope connected to a DFC450 camera equipped with Application Suite v 4.13.0 for Windows (Leica Microsystem, CMS GmbH, Switzerland).

### Electron microscopy

EDL muscles were dissected from mice, fixed at room temperature with 3.5% glutaraldehyde in 0.1 M NaCaCo buffer (pH 7.2), and kept at 4 °C in fixative until further use. Fixed muscles were then post-fixed, embedded, stained en-block, and sectioned for EM, as described previously [37]. For EM, ultrathin sections ( ~ 50 nm), were examined after staining in 4% uranyl acetate and lead citrate, with a Morgagni Series 268D electron microscope (FEI Company, Brno, Czech Republic), equipped with Megaview III digital camera (Munster Germany) at 60 kV.

### EM quantitative analysis

Estimates of the relative fiber volume occupied by myofibrils were calculated in transversal sections by the well-established stereology point counting technique [52, 53]. Briefly, 2 micrographs from each fiber (sample size: 15 fibers for each group) were taken at a magnification of 22 K excluding nuclei and subsarcolemmal regions. A grid of an orthogonal array of dots at a spacing of 0.35 μm was superimposed on each micrograph. The relative fiber volume occupied by the myofibrils vs. intermyofibrillar elements was then calculated by dividing the total number of dots by the number of dots falling in either one of the compartments.

### Behavioral testing

In the grip strength test, a peak amplifier is used to automatically measure the maximum force exerted by the animal’s forelimbs [54].

### Muscle mechanical analyses

EDL muscle from WT or CrT^−/y^ mice (PND40) was dissected under a stereomicroscope and mounted horizontally between the lever of a motor/force transducer system (305C, Aurora Scientific Inc.) and a lever carried by a micromanipulator in a trough, containing physiological Krebs–Henseleit solution (composition (mM): 119 NaCl, 4.7 KCl, 1.0 MgSO_4_, 25 NaHCO_3_, 1.2 KH_2_PO_4_, and 1.1 glucose), continuously saturated with carbogen (95% O_2_ and 5% CO_2_, pH 7.4) at room temperature (22–24 °C). The muscle was straightened just above its slack length using the micromanipulator. The trough was sealed with a Perspex cover and mounted vertically on the stage with the motor/force transducer system on the top. Trains of stimuli of alternate polarity to elicit fused tetani (frequency 100–120 Hz) were delivered by means of two platinum wire electrodes running parallel to the muscle, 1 cm apart. The intensity of the stimuli was increased until the isometric plateau force reached a maximum constant value, *T*_0_ (indicating that all cells in the muscle were activated). Muscle length was further finely adjusted using the micromanipulator to obtain the maximum isometric force corresponding to the plateau of the force–length relation. Isometric force was normalized to muscle Cross-Sectional Area (CSA), calculated from the muscle wet weight (w) according to the relation: CSA = (*w* · cosθ)/(*L*_f_ · δ), where θ is the pennation angle of the fibers (8,3°, [55]), *L*_f_ is the fiber length (cm), and δ is the muscle density, 1.056 g/cm^3^ [56]. *L*_f_ was obtained by multiplying the muscle length by 0.51 [55]. The force–velocity relation was determined by measuring the velocity of steady shortening (*V*) after a drop in force from *T*_0_ to a preset value *T* < *T*_0_ (see Supplementary Fig. 1A for the protocol). The force–velocity points were fitted to the Hill hyperbolic equation [56]: (*T* + *a*)x(*V* + *b*)=(*V*_0_ + *b*)x*a*, where *a*, *b*, and *V*_0_ (unloaded shortening velocity) are the regression parameters. Force and motor positions were recorded with a multifunction I/O board (PCI-6110E, National Instruments), and a dedicated program written in LabView (National Instruments) was used for signal recording and analysis.

#### RNA extraction, reverse transcription, and quantitative real-time PCR (RT-PCR)

Total RNA was extracted from EDL muscles using Trizol reagent, following the manufacturer’s instructions. The RNA was quantified using a Nanodrop spectrophotometer (Thermo Fisher Scientific). Complementary DNA (cDNA) was synthesized from 300 ng of total RNA using the SuperScript II cDNA Synthesis Kit with oligo(dT)12-18 primers for first-strand synthesis by reverse transcriptase. The cDNA was analyzed by quantitative real-time PCR (RT-PCR) on an IQ5 thermocycler using SYBR Green chemistry (Bio-Rad). Expression levels were quantified using the 2-ΔΔCT method [57].

Real-time PCR primer sequences were as follows:

MCU: FW AAAGGAGCCAAAAAGTCACG RV AACGGCGTGAGTTACAAACA MICU1: FW AAGGCAGCATCTTCTACAGCC RV CCTGCTCAAACTCCTCCATGT PGC-1α: FW CGCTGCTCTTGAGAATGGAT RV CGCAAGCTTCTCTGAGCTTC ACTIN: FW CTGGGCTCCTAGCACCATGAAGAT RV GGTGGACAGTGAGGCCAGGAT ATROGIN-1: FW GCAAACACTGCCACATTCTCTC RV CTTGAGGGGAAAGTGAGACG MUSA1: FW TCGTGGAATGGTAATCTTGC RV CCTCCCGTTTCTCTATCACG MURF1: FW CTTCTCTCAAGTGCCAAG RV CCTCAAGGCCTCTGCTATGT.

Raw data are included as Supplemental Material.

#### Western blotting and antibodies

Frozen muscles were pulverized using the QIAGEN Tissue Lyser for protein extraction. The protein lysis buffer used contained 50 mM Tris pH=7.5, 150 mM NaCl, 5 mM MgCl_2_, 1 mM DTT, 10% glycerol, 2% SDS, 1% Triton X-100, Roche Complete Protease Inhibitor Cocktail, 1 mM PMSF, 1 mM NaVO_3_, 5 mM NaF and 3 mM β-glycerophosphate. A total of 40 μg of protein, quantified using the BCA assay, was loaded onto 4–12% acrylamide gels for SDS-PAGE. Proteins were separated by SDS-PAGE and transferred onto nitrocellulose membranes using the semi-dry method. Blots were blocked for 1 h at room temperature with 5% non-fat dry milk in TBS-T buffer (50 mM Tris, 150 mM NaCl, and 0.01% Tween.20, pH = 7.4) and then incubated at 4 °C with primary antibodies overnight. Secondary antibodies were applied for 1 h at room temperature. The following primary antibodies, used at dilution 1:1000, were used: COX IV (Cell Signaling Technology, Cat #4844), MCU (Merck, Cat #HPA016480), MICU1 (Merck, Cat #HPA037479), Akt (pan) (C67E7) (Cell Signaling Technology, Cat #4691S), Phospho-Akt (Ser473) (D9E) (Cell Signaling Technology, Cat #4060S), S6 ribosomal protein (5G10) (Cell Signaling Technology, Cat #2217), Phospho-S6 (S240/244)XP (Cell Signaling Technology, Cat #5364), 4EBP1 (Cell Signaling Technology, #9452), phosphor-4EBP1 (Thr37/46) (Cell Signaling Technology, Cat #9459), Actin (Merk, Cat # A4700), SERCA1 (Thermo Fisher Scientific, Cat # MA3-911), SERCA2 (Thermo Fisher Scientific, Cat # 2A7-A1), AFG3L2 (Thermo Fisher Scientific, Cat # PA5-95347), TOM20 (Cell Signaling Technology, Cat #42406), PDH (Cell Signaling Technology, Cat #2784), Total-OXPHOS (Abcam, Cat # ab110413).

Full-length uncropped original western blots used in the manuscript are included as Supplemental Material.

#### FDB fiber dissociation and culture

FDB fibers were isolated from muscle digested in collagenase A (4 mg/mL) (Roche) dissolved in Tyrode’s salt solution pH = 7.4, containing 10% FBS. Single fibers were obtained by mechanical dissociation and plated on laminin-coated glass coverslips. The FDB fibers were cultured in DMEM with HEPES, supplemented with 10% FBS, containing penicillin (100 U/mL) and streptomycin (100 U/mL), at 37 °C with 5% CO_2_.

#### Cytosolic and mitochondrial [Ca^2+^] measurements in FDB fibers

For cytosolic [Ca^2+^] measurements, FDB fibers were loaded with 2 μM cytosolic Fura-2/AM (Thermo Fischer Scientific, #F1221) diluted in Krebs-Ringer modified buffer (135 mM NaCl, 5 mM KCl, 1 mM MgCl_2_, 20 mM HEPES, 1 mM MgSO_4_, 0.4 mM KH_2_PO_4_, 1 mM CaCl_2_, 5.5 mM glucose, pH = 7.4), containing 0.02% Pluronic acid for 20 min and then washed with Krebs-Ringer modified buffer in the presence of 75 μM N-benzyl-P-toluenesulfonamide (BTS) to avoid fiber contraction. Ca^2+^ release from the ER was induced with 20 μM caffeine treatment.

For mitochondrial [Ca^2+^] measurements, FDB fibers were loaded with 2 μM mitochondrially targeted mitoFURA-2/AM [58] diluted in Krebs-Ringer modified buffer (described above) containing 0.02% Pluronic acid for 30 min and then washed with Krebs-Ringer modified buffer in the presence of 75 μM BTS. Ca^2+^ release from the ER was induced with 20 μM caffeine treatment.

Cytosolic and Mitochondrial [Ca^2+^] experiments were performed on a Zeiss Axiovert 200 microscope equipped with a 40×/1.3 N.A. PlanFluor objective. Excitation was performed with a DeltaRAM V high-speed monochromator (Photon Technology International) equipped with a 75 W xenon arc lamp. Images were captured with a high-sensitivity Evolve 512 Delta EMCCD (Photometrics). The system was controlled by MetaMorph 7.5 (Molecular Devices) and was assembled by Crisel Instruments. Images were collected by alternatively exciting the fluorophore at 340 and 380 nm and fluorescence emission was recorded through a 515/30 nm band-pass filter (Semrock). Image analysis was performed with Fiji distribution of the ImageJ software. The images analyzed were background subtracted.

Mitochondrial Ca^2+^ efflux was normalized on the maximum Ca^2+^ value after caffeine stimulation.

#### Mitochondrial membrane potential

Isolated FDB muscle fibers were incubated with 20 nM tetramethyl rhodamine methyl ester dye (TMRM) in non-quenching mode together with 75 μM BTS in Krebs-Ringer modified buffer for 20 min at 37 °C. In non-quenching mode, the probe is used at lower concentrations to avoid dye aggregation and quenching within mitochondria. Consequently, depolarized mitochondria will accumulate less of the cationic dye and display reduced fluorescence, whereas hyperpolarised mitochondria will accumulate more dye, resulting in higher fluorescence [38]. Data acquisition was done with Leica TCS-SP5-II equipped with a PlanApo 100×/1.4 N.A. objective. The TMRM fluorescence was excited by the 543 nm HeNe laser, and its emission was collected in the 555-700 nm range. 10 μM carbonyl cyanide 3-chlorophenylhydrazone (CCCP) was added at the end of the experiment to completely collapse the Δψ. Data are expressed as difference of background corrected TMRM fluorescence before and after CCCP depolarization.

#### Muscle ATP extraction and quantification

ATP was extracted from EDL muscles from either WT or CrT^−/y^ mice using the phenol-chloroform method, as previously reported [59]. ATP measurements were performed with the ATP Determination Kit (Thermo Fisher Scientific, Catalog number: A22066), following the manufacturer’s instructions. Results were background corrected and normalized for muscle weight.

#### Statistical analysis of data

All data were analyzed for statistical significance using GraphPad Prism software. Statistical data are presented as mean ± SEM. Depending on the experiments, a Student’s *t*-test, Mann-Whitney t-test, and one or two-way ANOVA with Bonferroni post hoc test were applied. p < 0.05 was considered significant.

## Supplementary information

Supplemental Material

Supplementary information

## Data Availability

The data generated or analyzed during this study are accessible upon reasonable request from the corresponding author.
