# Supplementary material for: Creatine transporter (SLC6A8) knockout mice exhibit reduced muscle performance, disrupted mitochondrial Ca2+ homeostasis, and severe muscle atrophy
Source: Cell Death Dis. 2025 Feb 14;16(1):99. doi: 10.1038/s41419-025-07381-x (PMC11828924; doi:10.1038/s41419-025-07381-x)
Supplement: Supplementary file 1 — Supplemental Material [file 41419_2025_7381_MOESM1_ESM.pdf]

Supplementary Figures

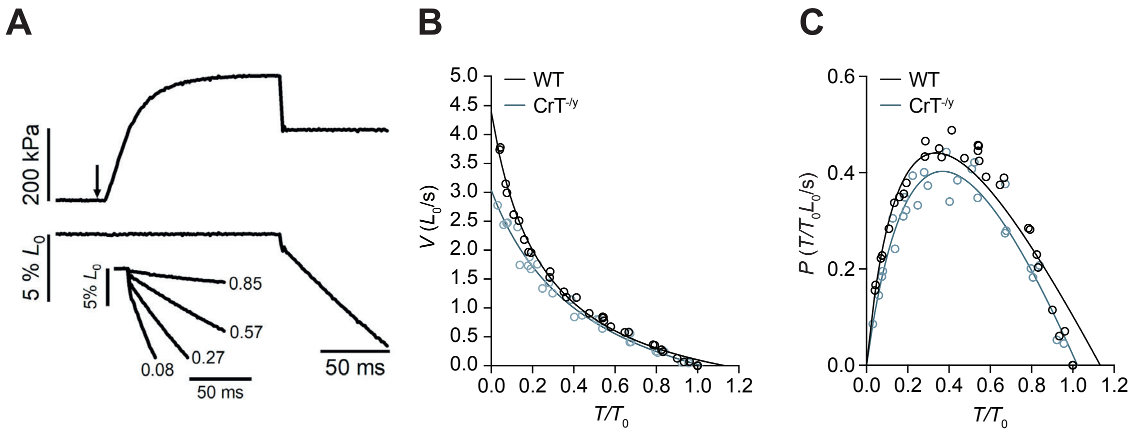

Supplementary Figure 1. (A) Mechanical protocol. Sample records of length change (lower traces) during isotonic contraction against a load of 0.5 T_0 (upper trace) from EDL muscle of WT mouse. The vertical line indicates the stimulus start. Inset, sample records of length changes during isotonic contractions against different loads as indicated by the values close to the traces. **(B)** and **(C)** Same relations of Fig. 2B and C respectively with force expressed in T/T_0.

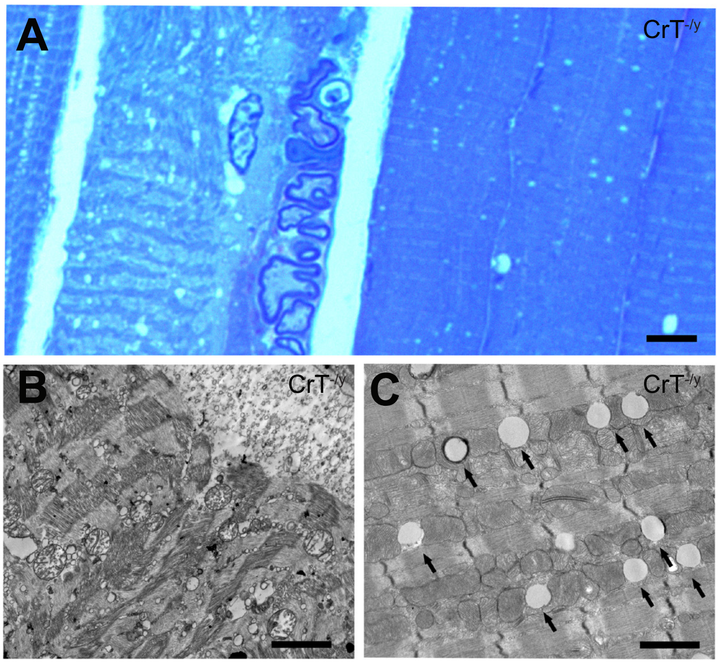

Supplementary Figure 2. Representative images from light (toluidine Blue-stained) and electron microscopy of EDL fibers showing additional abnormalities in CrT$^{-/y}$ mice. In CrT$^{-/y}$ mice about 35% of the fibers are severely damaged with disruption of the sarcomeric cross-striation in large area of the fiber as shown in light (**A**, left fiber) and in electron (**B**) microscopy images. Several EDL fibers in CkT$^{-/y}$ mice show an unusual high number of lipid droplets (A and **C**, black arrows).

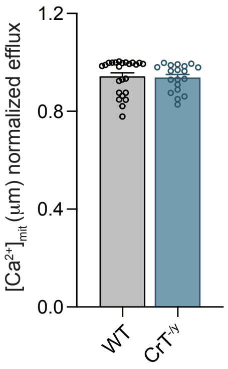

Supplementary Figure 3. Mitochondrial Ca^{2+} efflux normalized on maximum Ca^{2+} value after caffeine stimulation.

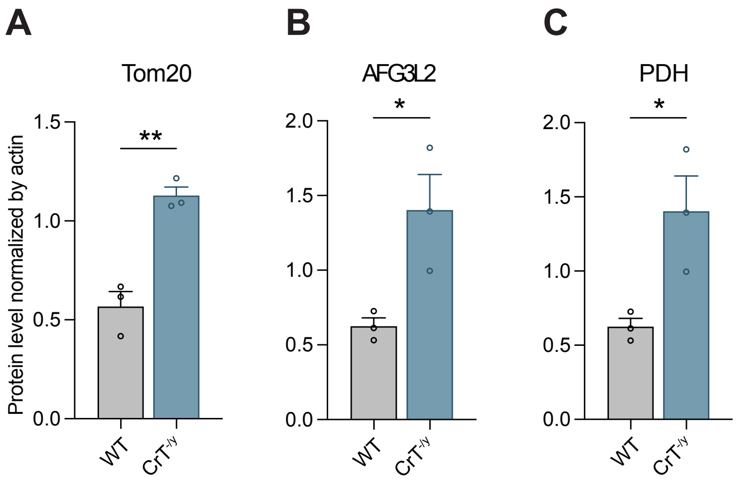

Supplementary Figure 4. (**A**) – (**C**) Quantification of the immunoblots showed in Fig. 5L. The levels of the protein were normalized by actin levels. Data are presented as mean ± SEM. n=3. For data analysis, parametric Student t-test (two-tailed, unpaired) was used. * $p<0.05$; ** $p<0.01$.
