# Supplementary material for: Creatine transporter (SLC6A8) knockout mice exhibit reduced muscle performance, disrupted mitochondrial Ca2+ homeostasis, and severe muscle atrophy
Source: Cell Death Dis. 2025 Feb 14;16(1):99. doi: 10.1038/s41419-025-07381-x (PMC11828924; doi:10.1038/s41419-025-07381-x)
Supplement: Supplementary file 2 — Supplementary information [file 41419_2025_7381_MOESM2_ESM.pdf]

Figure 2

F

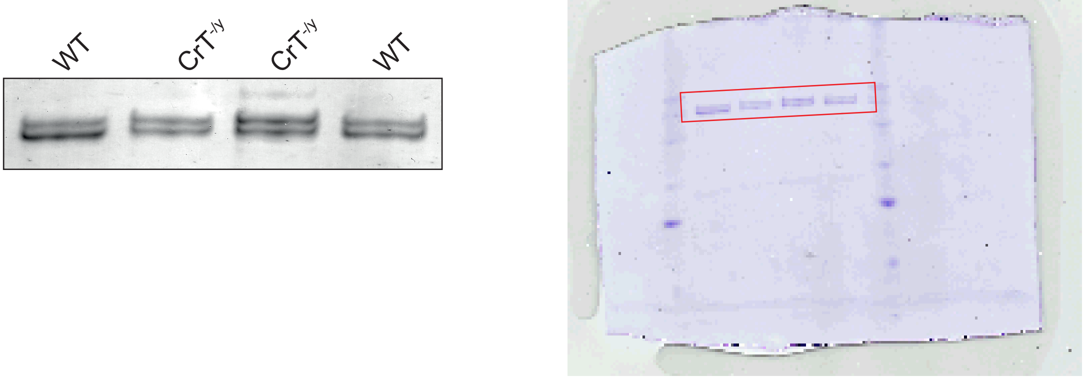

Figure 3

D

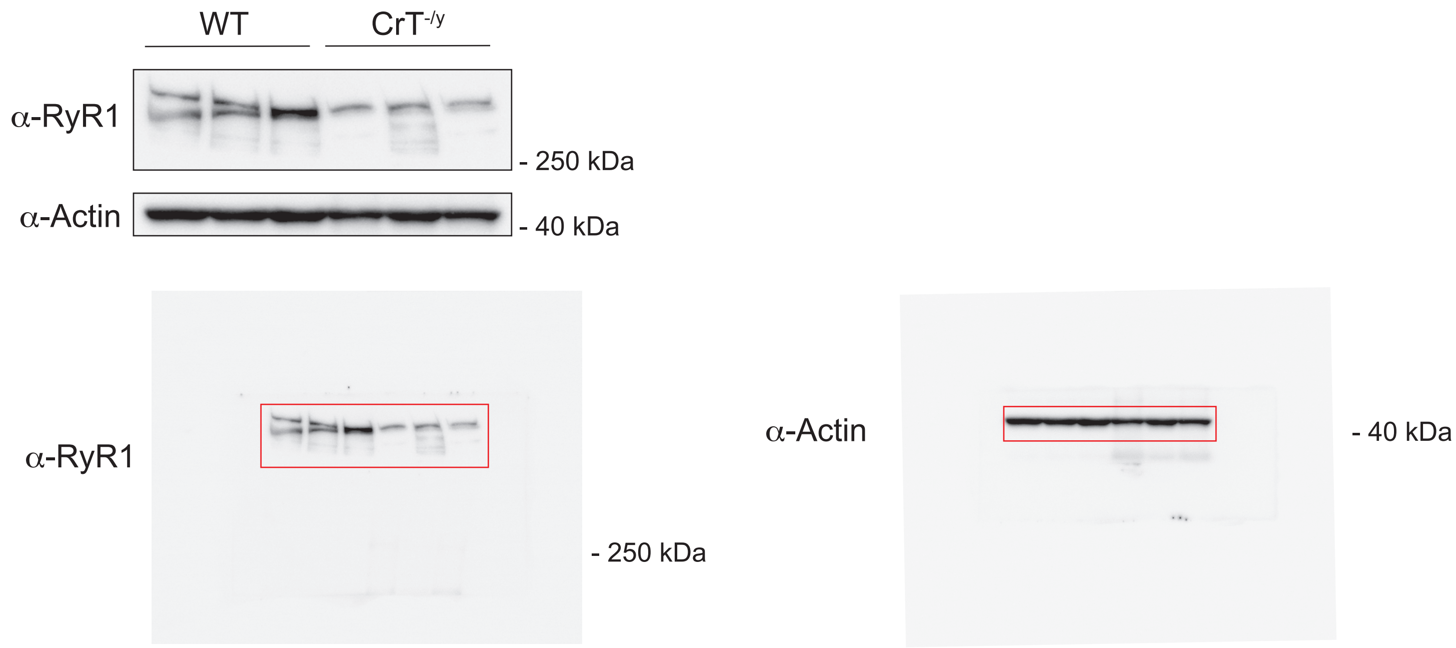

F

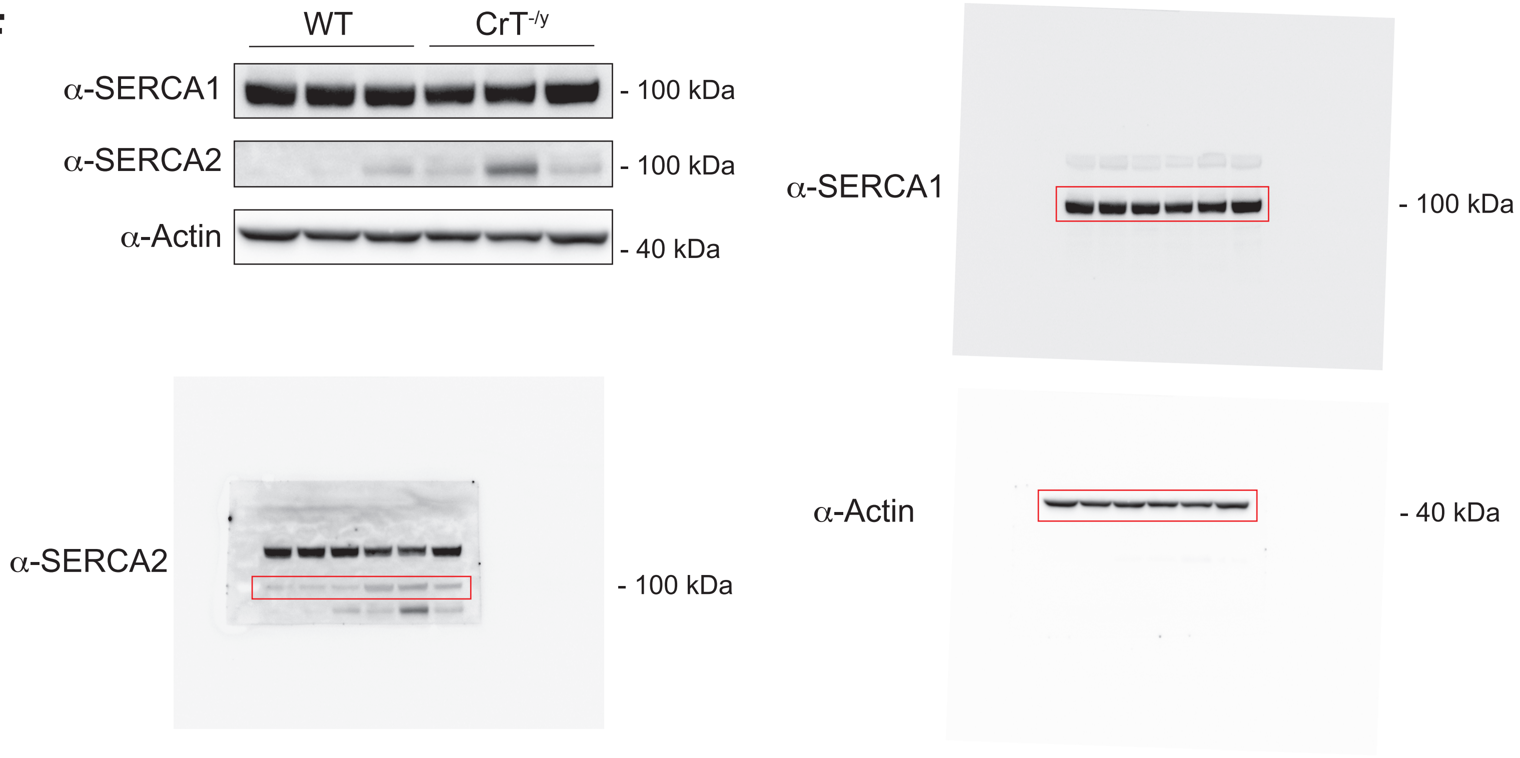

Figure 5

F

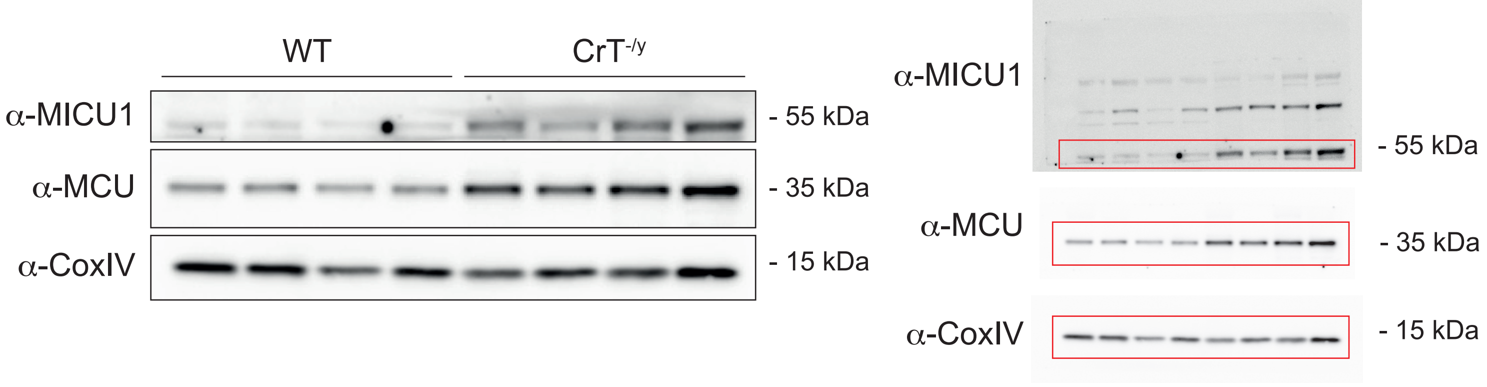
WT
CrT-/y
α-MICU1
- 55 kDa
α-MCU
- 35 kDa
α-CoxIV
- 15 kDa
α-MICU1
- 55 kDa
α-MCU
- 35 kDa
α-CoxIV
- 15 kDa

H

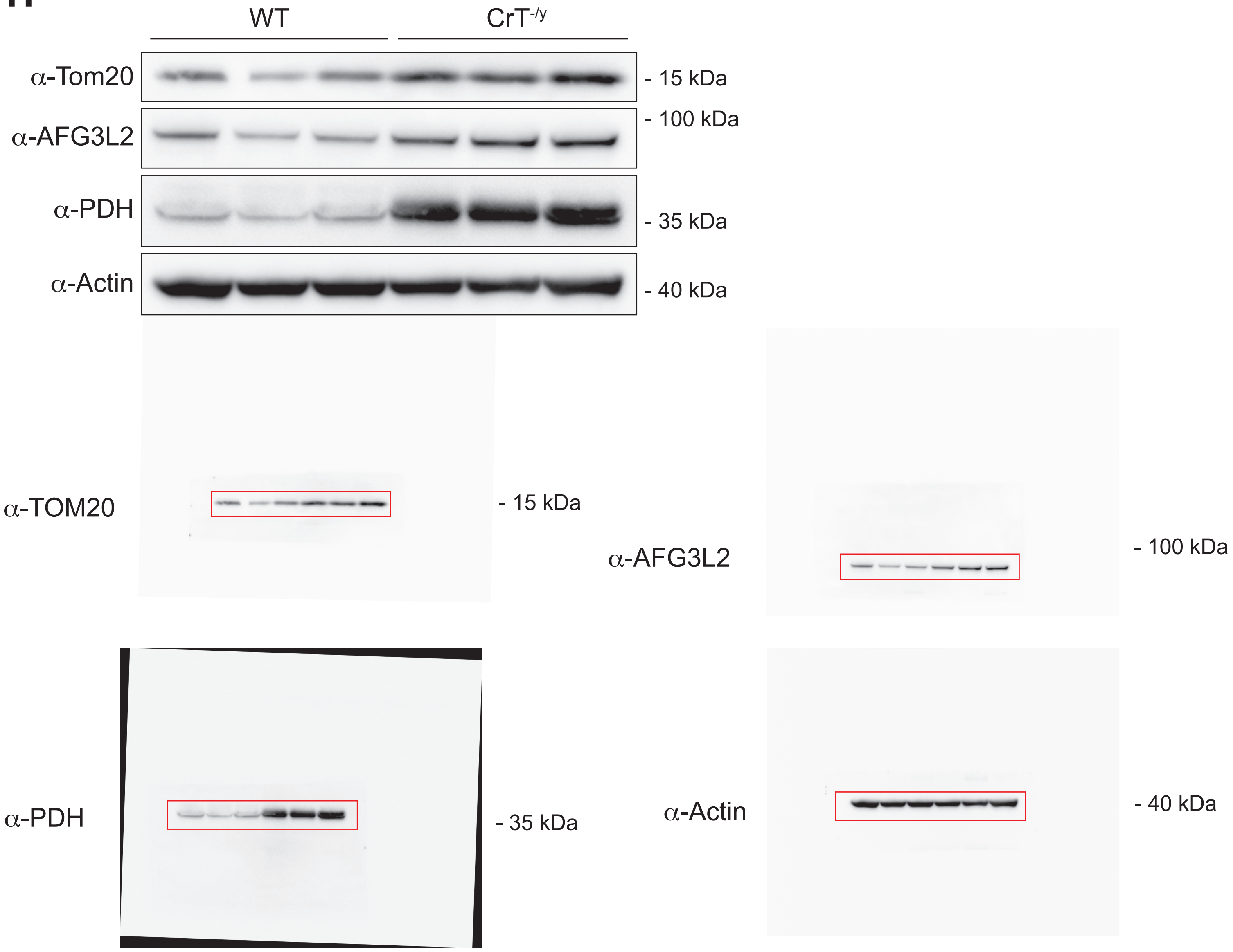
WT
CrT-/y
α-Tom20
- 15 kDa
α-AFG3L2
- 100 kDa
α-PDH
- 35 kDa
α-Actin
- 40 kDa
α-TOM20
- 15 kDa
α-AFG3L2
- 100 kDa
α-PDH
- 35 kDa
α-Actin
- 40 kDa

Figure 5

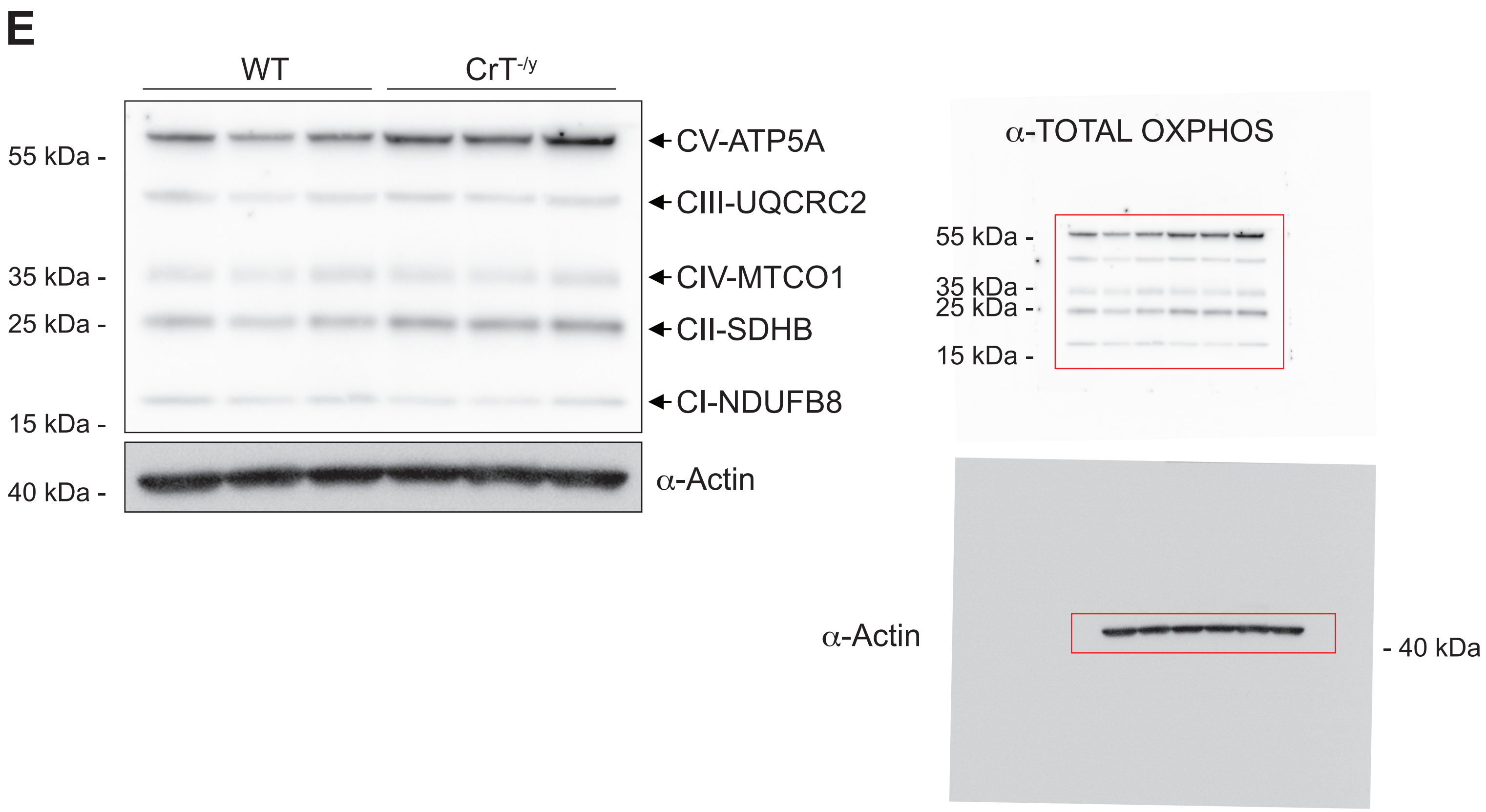

Figure 6

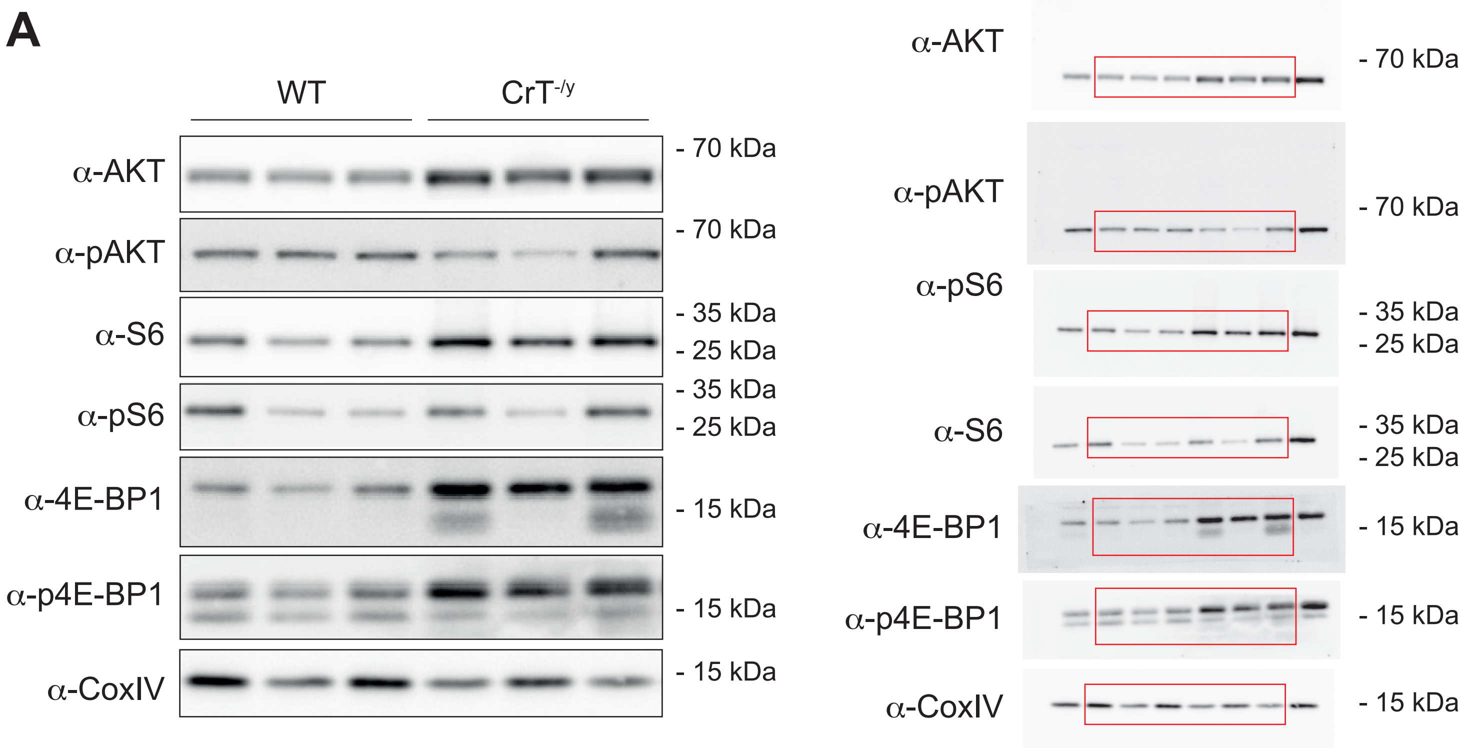

qPCR data

Figure 5G

	**MCU**	**MICU1**
Sample	Expression ratio	Expression ratio
WT 1	0.835697724	0.978771604
WT 2	1.248329053	1.037129149
WT 3	0.915973223	0.984099247
$CrT^{-/y}$ 1	2.071035926	3.252158027
$CrT^{-/y}$ 2	1.314156335	2.096260642
$CrT^{-/y}$ 3	1.663386829	1.659007486

Figure 5I

	**Pgc-1α**
Sample	Expression ratio
WT 1	0.981710603
WT 2	0.863260838
WT 3	1.279378492
WT 4	0.815851207
WT 5	1.129587977
WT 6	1.000793352
$CrT^{-/y}$ 1	1.44774111
$CrT^{-/y}$ 2	2.120125856
$CrT^{-/y}$ 3	1.839987608
$CrT^{-/y}$ 4	1.486664988
$CrT^{-/y}$ 5	2.105324053

Figure 6E

	**Atrogin-1**	**MuRF-1**	**MUSA1**
Sample	Expression ratio	Expression ratio	Expression ratio
WT 1	1.010450136	0.704101395	1.086560261
WT 2	1.306141124	0.508806614	0.925527601
WT 3	1.183194273	1.050788549	1.012877918
WT 4	0.963576924	1.49522963	0.871966065
WT 5	0.901401912	1.483196354	1.247955679
WT 6	0.737282772	1.197816172	0.902196129
$CrT^{-/y}$ 1	2.567115313	0.895972561	2.12008118
$CrT^{-/y}$ 2	4.807150752	1.113741371	2.94222575
$CrT^{-/y}$ 3	2.547160189	1.346723092	3.79361092
$CrT^{-/y}$ 4	1.905501117	0.987358768	2.470729545
$CrT^{-/y}$ 5	3.952746645	1.500608852	4.098894542
